# Mapping Bullous Emphysema With Lung Ultrasound: A Prospective Multicentre Study

**DOI:** 10.1111/resp.70021

**Published:** 2025-03-09

**Authors:** Kinan El Husseini, Thomas Flament, Sophie Laroumagne, Damien Basille, Mathilde Le Brun, Elise Noël‐Savina, Philippe Richard, Gilles Mangiapan, Elise Artaud‐Macari

**Affiliations:** ^1^ Department of Pneumology Thoracic Oncology and Respiratory Intensive Care Unit Rouen France; ^2^ Department of Pneumology A CHU Bichat, APHP Paris France; ^3^ G‐ECHO: Lung Ultrasound Working Group of the French Society of Respiratory Diseases (SPLF) Paris France; ^4^ Department of Respiratory Medicine CHU Tours Tours France; ^5^ Department of Thoracic Oncology Pleural Diseases and Interventional Pulmonology, AP‐HM Marseille France; ^6^ Department of Pneumology and Critical Care Unit CHU Amiens‐Picardie Amiens France; ^7^ Department of Pneumology Larrey Hospital, CHU Toulouse Toulouse France; ^8^ Department of Pneumology Centre Hospitalier Intercommunal de Créteil Créteil France; ^9^ Normandie Univ, UNIROUEN, UR3830, CHU Rouen Rouen France

**Keywords:** COPD, emphysema, pneumothorax, radiology and other imaging

## Abstract

**Background and Objectives:**

Lung ultrasound holds high diagnostic performance for pleural diseases, notably pneumothorax. Bullous emphysema is a potential differential diagnosis of pneumothorax on ultrasound, but its precise semiology is poorly known. This study aimed to delineate the sonographic presentation of bullous emphysema and assess the diagnostic performance of common ultrasound features in identifying bullae.

**Methods:**

From June 2019 to June 2021, patients with CT scanner‐confirmed bullous emphysema were prospectively included. Investigators performed a standardised 14‐region lung ultrasound. Sonographic features of bullous and non‐bullous regions were compared. Diagnostic performances for bullae were calculated for each sign, and an additive score was constructed using signs with specificity > 85%. Pearson's correlation was used to examine the relationship between this score, bulla size, and respiratory functional parameters.

**Results:**

Thirty‐six patients were included, mostly male (*n* = 33 patients, 91.7%), with an average age of 62 ± 11 years. Bullae mostly affected apical regions (*n* = 24 patients, 67%). Bullous regions displayed a more frequent absence of lung sliding (34% vs. 11% in non‐bullous regions, *p* < 0.01), barcode sign (15% vs. 3%, p < 0.01), increased A‐line visibility (16% vs. 8%, *p* = 0.048), and absence of Z lines (62% vs. 44%, *p* = 0.018). A bulla‐point sign was visualised in 4% of bullous regions. Absent lung sliding was more frequent in patients with pulmonary distension and in apical regions. Patient bulla score (3 [2–6]) correlated with bulla size (*r* = 0.53 [0.25;0.73], *p* < 0.001), FEV_1_ (*r* = −0.38 [−0.60;−0.03], *p* = 0.022), and forced vital capacity (*r* = −0.38 [−0.64;−0.08], *p* = 0.021).

**Conclusion:**

Our findings challenge previous data about the specificity of ultrasound signs of pneumothorax in patients with bullous emphysema, highlighting the need for cautious interpretation in clinical practice.

**Trial Registration:** NCT04012359

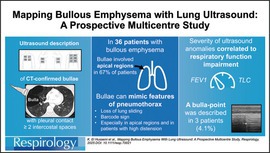

## Graphical abstract

**See related** article

**Summary**

- This study confirms the existence of the ‘bulla‐point’, a sonographic sign echoing the lung‐point associated with pneumothorax.
- Other signs of pneumothorax such as absent lung sliding or barcode sign are also prevalent in bullous emphysema, especially in patients with high pulmonary distension or in apical regions.

## 1 Introduction

Chronic obstructive pulmonary disease (COPD) poses a significant strain on healthcare systems worldwide [1]. It is characterised by persistently obstructed airflow due to diffuse airway inflammation and irreversible destruction of the distal airways [2]. A major but variable component of COPD is emphysema, defined as the abnormal, permanent enlargement of airspaces distal to terminal bronchioles and the destruction of alveolar walls without fibrosis [3]. This condition typically appears as centrilobular emphysema, predominantly in the upper lobes where airborne irritants, like tobacco smoke, catalyse the destruction of the alveolar walls [4, 5].

Progression of COPD is closely linked to the worsening of emphysema [6], with destroyed airspaces sometimes conflating in large bullae. Bullae are defined on chest computed tomography (CT) as avascular, thin‐walled hypodensities, larger than 1 cm in diameter [7]. When sufficiently large, these bullae can impede lung function through hyperinflation mechanisms, reaching the stage of “giant bullae” or “vanishing lung syndrome” when they occupy more than a third of a hemithorax [8, 9, 10]. These conditions are often misdiagnosed as pneumothorax, as their appearance can be similar on chest radiography [11, 12, 13]. In addition, spontaneous pneumothorax can complicate severe, bullous emphysema [14]. Therefore, accurately differentiating pneumothorax and bullae is crucial in the setting of acute respiratory failure in emphysematous patients, as misdiagnosis leading to invasive drainage of a bulla can result in potentially disastrous iatrogenic pneumothorax or hemothorax [15].

Lung ultrasound (LUS) is a non‐invasive bedside test with growing high‐quality evidence showing high performance in the management of pulmonary diseases. LUS‐guided thoracocentesis improves the procedure success rate and reduces complication risk [16], while point‐of‐care LUS among patients with suspected pneumothorax exhibits excellent diagnostic performance approaching CT and far exceeding chest radiography [17, 18, 19], with well‐defined diagnostic criteria. LUS also shows promising results in the study of parenchymal disease such as pneumonia and interstitial disease. Guidelines from multiple scientific societies therefore incorporate LUS as a vital part of routine care, from the monitoring of mechanical ventilation and fluid administration in intensive care settings to the bedside management of pleural effusions and pneumothorax [20, 21, 22, 23]. However, some studies have reported that bullous emphysema could mimic pneumothorax on LUS [24, 25, 26, 27], with signs previously considered highly specific for pneumothorax also appearing in patients with bullous emphysema [24, 28]. This overlap may decrease the diagnostic performance of LUS in emphysematous patients, given that the precise sonographic features of bullous emphysema have not been systematically reported.

Thus, our study aimed to provide a comprehensive description of LUS findings in patients with bullous emphysema using a standardised method. This analysis will contribute to refining the understanding of LUS semiology in the context of bullous emphysema, with a focus on those signs previously linked more exclusively to pneumothorax.

## 2 Methods

A prospective observational study was performed over a 24‐month period from June 2019 to June 2021 at four French tertiary centres. The study design received approval from a national ethics committee (CPP, registry number 2019T3‐06 HPS [2018‐A00497‐48]).

### 2.1 Study Population and Protocol

**FIGURE 1:**
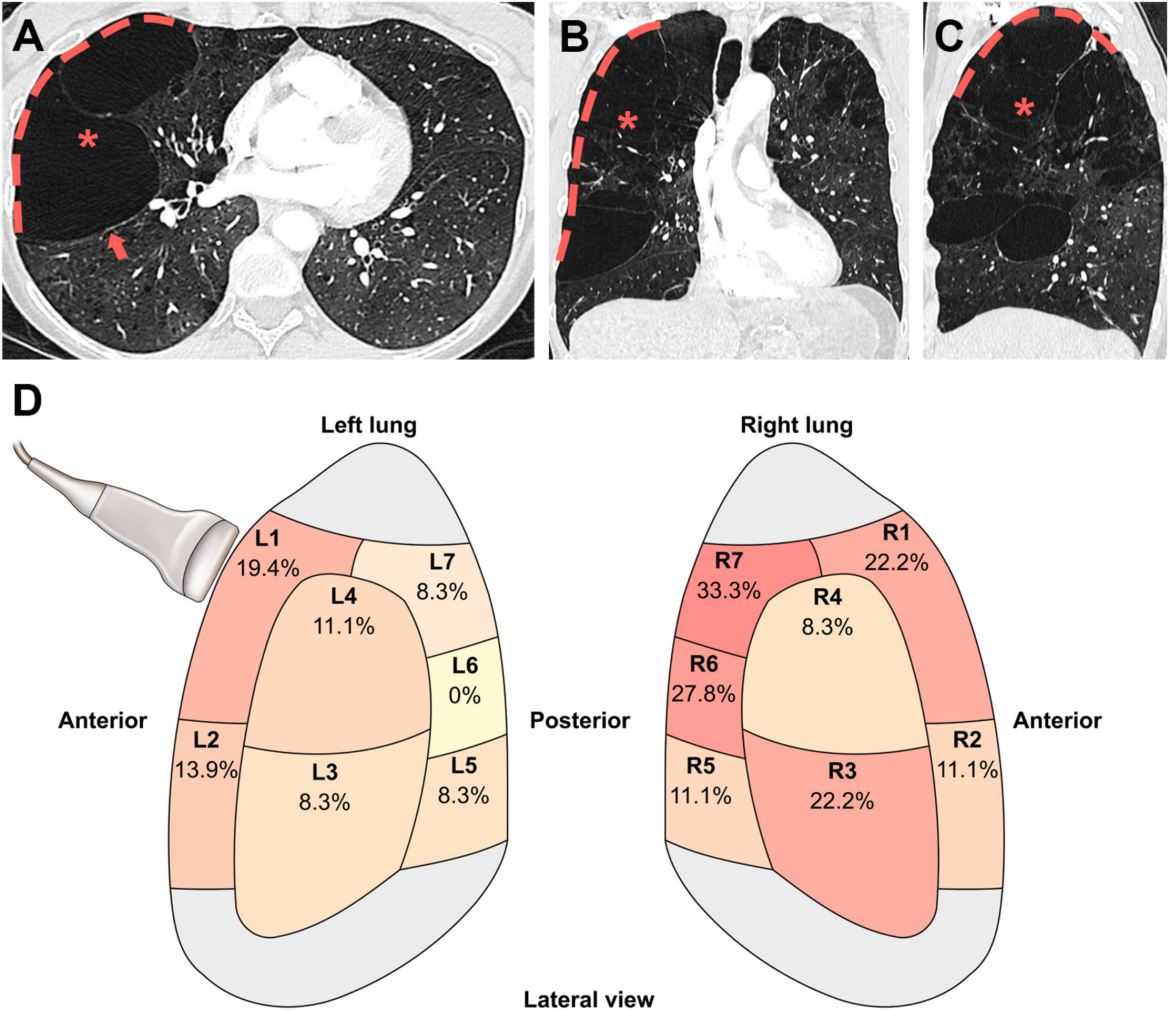
Bullous emphysema CT aspect and topography in the study population. (A–C) Representative chest CT sections (parenchymal window) of a voluminous bullous dystrophy (asterisk) with pleural contact (dotted line), with reconstruction in the axial (A), coronal (B) and sagittal (C) planes. Arrows indicate bullous wall, consisting of destroyed and compacted parenchyma. (D) Topography of the most voluminous bullae with pleural contact in the study population, according to CT scan. Each area is shown in lateral view on the surface of the right and left lungs (L1–L7 on the left, R1 to R7 on the right). Within each area, the percentage of included patients whose bullous emphysema with pleural contact involves that area is shown.

This study included patients referred in an outpatient or programmed inpatient setting, with a pre‐existing COPD diagnosis and bullous emphysema identified on chest CT. As bullous emphysema is irreversible and its natural mode of evolution in the absence of specific therapeutic interventions is an increase in size or, at best, stability, CT could be performed up to 2 years prior to inclusion, and repeat examination on inclusion visit was not mandatory. Bullae were defined according to the Fleischner society description of avascular low‐attenuation areas > 1 cm in diameter, with a thin but perceptible wall [5, 7]. Patients were included if bullae were present on chest CT, and bullae walls were in direct contact with the pleura along at least 2 full intercostal spaces, as measured on the coronal or sagittal views (an example is shown on Figure 1B,C). Exclusion criteria were past medical history of pleurodesis, pleural plaques, or pachypleuritis homolateral to bullous emphysema, pulmonary resection, lung volume reduction surgery or endoscopic lung volume reduction performed after the most recent chest CT, pneumothorax occurring after the most recent chest CT, current acute hemodynamic, respiratory or neurological failure, recent thoracic surgery (less than 7 days prior), or subcutaneous emphysema.

During a single visit, each participant underwent a standard medical examination, chest radiography, bedside LUS, and pulmonary function tests. Chest radiography was reviewed to verify the absence of abnormalities that could interfere with the LUS examination, particularly condensations and pleural effusions. A physician accredited in thoracic sonography performed the LUS. Probe choice (convex or linear) was left to the sonographer appreciation, and both could be used during the same examination. Results were recorded using a standardised form (Figure S1).

### 2.2 Lung Topographical Segmentation and Lung Ultrasound

Lungs were anatomically divided into 7 numbered regions, bilaterally [29]; region 1 (apico‐anterior), 2 (anterior), 3 (axillar inferior), 4 (axillar superior), 5 (postero‐inferior), 6 (posterior), and 7 (apico‐posterior). In each region, investigators recorded the following findings (finding [possible values]): in B mode, lung sliding (present/absent), B lines (number per field of view), Z lines (present/absent), A lines visibility (normal/increased), lung pulse (present, absent), lung point, that is, the visualisation in B mode of a “break” interrupting the pleural sliding which marks the junction between normal and decreased lung sliding; in time‐movement (TM) mode, general aspect (seashore/barcode).

Lung sliding is a to‐and‐fro movement of the pleural line, translating into a seashore sign in TM mode, and a barcode sign when it is absent, as is the case in pneumothorax [30]. A‐lines are horizontal linear artefacts corresponding to repetitions of the pleural line, with increased visibility in pneumothorax, while B and Z lines are vertical linear artefacts often seen in normal lung or interstitial disease, and absent in pneumothorax [31]. Lung point is a sign sometimes seen at the edges of a pneumothorax, which can be used to quantify its abundance. It corresponds to the interface between pneumothorax and healthy lung. In this area, in B‐mode, healthy lung produces a sliding pleuro‐pulmonary line, mobile with respiratory movements. On the other side of the interface, an immobile pleural line is observed, producing a barcode aspect in TM‐mode [32, 33]. Bulla‐point sign, sometimes referred to as bleb‐point, is a sign mimicking the lung point of pneumothorax outside of the context of pneumothorax [24, 26]. Lung pulse corresponds to the association of absent lung sliding with the perception of heart activity at the pleural line, which is present in the case of atelectasis, or physiologically during apnoea [34]. There were one to two sonographers in each centre. Every sonographer had completed specific training in lung ultrasound techniques, and routinely practised LUS at their institution.

Images and videos were saved in both modes for all regions, annotated with side and region number. Supplementary findings could also be recorded, and additional images could be saved. The topography of the most voluminous bullae was also collected using the most recent CT scan available, following the same topographical nomenclature as the LUS. Regions affected by bullae with pleural contact were defined as “bullous regions”

### 2.3 Statistical Analysis

Variables presented in descriptive analyses are presented as either median [inter‐quartile range] or mean (standard deviation) for quantitative variables and as absolute and relative frequencies for qualitative variables. Comparisons of quantitative and qualitative variables were made using the Mann–Whitney test and chi‐squared or Fisher exact test, respectively. Diagnostic parameters for identifying bullae were calculated for each sonographic feature. An additive score was constructed using the most specific signs (specificity > 85%) and calculated in every lung region, summed for each participant (total patient bulla score). The Shapiro‐Wilk test was used to analyse the normality of data. Pearson's correlation was used to examine the relationship between quantitative variables. All tests were two‐sided, with *p* = 0.05 indicating statistical significance. Adjustment for multiple comparisons was performed using the adaptive Benjamini‐Hochberg test with a false discovery rate set at 5%. Statistical calculations were performed using the R environment (R Statistical Software, version 4.0.5; R Foundation for Statistical Computing, Vienna, Austria).

Additional details on clinical and paraclinical information collected from the electronic medical records, CT image acquisition and analysis, and lung ultrasound machines used are reported in the Supporting Information (Supporting Information Methods).

## 3 Results

### 3.1 Demographic Characteristics

This study included 36 patients in 4 centres (*n* = 18, 11, 5 and 2, respectively). Patients were mostly male (*n* = 33, 91.7%), with a mean age of 62.1 ± 10.6 years, significant tobacco exposure (48 ± 22 pack‐year). Seven (19.4%) patients had a past medical history of pneumothorax. Chest radiography performed on the day of inclusion showed no significant parenchymal or pleural abnormalities. Pulmonary function tests showed, on average, an obstructive ventilatory disorder (Forced expiratory volume in 1 s (FEV_1_): 54.2%pred. ± 25.0%pred.), lung distension (Total Lung Capacity (TLC): 116.0%pred. ± 18.3%pred., Residual Volume (RV): 172.4% ± 63.2%), and severe diffusion impairment (Diffusion Lung Capacity of Carbon Monoxide (TLCO): 47.8%pred. ± 17.5%pred.). Twelve (33.3%) patients were on long‐term oxygen therapy and 6 (16.7%) on home non‐invasive ventilation. Respiratory rehabilitation was ongoing or already performed in 11 (30.6%) patients (Table 1).

**TABLE 1:** Demographic and clinical characteristics of the study population.

Variable	Total population (*n* = 36)
Age, years	62.1 ± 10.6
Sex	
Male, *n* (%)	33 (91.7)
Female, *n* (%)	3 (8.3)
Body‐mass index, kg/m^2^	22.2 ± 3.5
Tobacco exposure	
Current or previous smoker, *n* (%)	36 (100)
Exposure, pack‐years	48 ± 22
Past cardio‐pulmonary history	
COPD diagnosis, *n* (%)	35 (97.2)
Pneumothorax, *n* (%)	7 (19.4)
Thoracic surgery, *n* (%)	8 (22.2)
< 1 month, *n* (%)	1 (2.8)
> 1 month, *n* (%)	7 (19.4)
Other^a^, *n* (%)	12 (33.3)
Respiratory clinical examination	
Decreased lung sounds, *n* (%)	24 (66.7)
Abolished lung sounds, *n* (%)	3 (8.3)
Pharmacological treatment	
Short‐acting beta‐agonist, *n* (%)	15 (41.7)
Long‐acting beta‐agonist, *n* (%)	25 (69.4)
Long‐acting muscarinic agent, *n* (%)	23 (64.0)
Inhaled corticosteroids, *n* (%)	18 (50.0)
Azithromycin, *n* (%)	1 (2.8)
Systemic corticosteroids, *n* (%)	2 (5.6)
Breathing apparatus	
Long‐term oxygen therapy, *n* (%)	12 (33.3)
Continuous positive airway pressure, *n* (%)	3 (8.3)
Home noninvasive ventilation, *n* (%)	6 (16.7)
Respiratory rehabilitation	
Performed, *n* (%)	5 (13.9)
Planned, *n* (%)	6 (16.7)
Pulmonary function testing	
FEV_1_, L	1.7 ± 0.9
FEV_1_, %th	54.2 ± 25.0
FVC, L	3.1 ± 1.1
FVC, %th	81.0 ± 21.3
TLC, %th	116.0 ± 18.3
RV, %th	172.4 ± 63.2
FEV_1_/FVC, %	52.3 ± 15.2
TLCO, %	47.8 ± 17.5
KCO, %	54.4 ± 19.5

Abbreviations: BE, bullous emphysema; COPD, chronic obstructive pulmonary disease; FEV_1_, forced expiratory volume in 1 s; FVC, forced vital capacity; KCO, carbon monoxide transfer coefficient; RV, residual volume; TLC, total lung capacity; TLCO, diffusion lung capacity of carbon monoxide.

^a^ Other cardio‐pulmonary history: obstructive sleep apnoea 3 (8.3%), pulmonary aspergillosis 4 (11.1%), pulmonary tuberculosis 1 (2.8%), lung cancer 1 (2.8%), interstitial lung disease 1 (2.8%), asthma 1 (2.8%), α‐1‐antitrypsin deficiency 1 (2.8%).

On chest CT, the bullae affected 2.0 ± 1.2 lung regions per patient (corresponding to 3.8 ± 2 intercostal spaces) and involved predominantly apical regions (24 cases, 66.7%) (Table 2).

**TABLE 2:** Bullae characteristics on thoracic CT.

Variable	Total population (*n* = 36)
Interval between thoracic CT and LUS, days	14 [0; 46]
Pleural contact of bullae walls, intercostal spaces	3.78 ± 2.07
Side	
Left, *n* (%)	12 (33.3)
Right, *n* (%)	24 (66.6)
Topographical regions affected, *n* (%)	
R1/L1	15 (41.7)
R2/L2	9 (25.0)
R3/L3	11 (30.6)
R4/L4	7 (19.4)
R5/L5	7 (19.4)
R6/L6	10 (27.8)
R7/L7	15 (41.7)

Abbreviation: CT, computerised tomography.

Typical bullous emphysema CT images, as well as an anatomical mapping of bullous regions in the study population, are represented in Figure 1.

### 3.2 Sonographic Semiology of Bullous Emphysema

**FIGURE 2:**
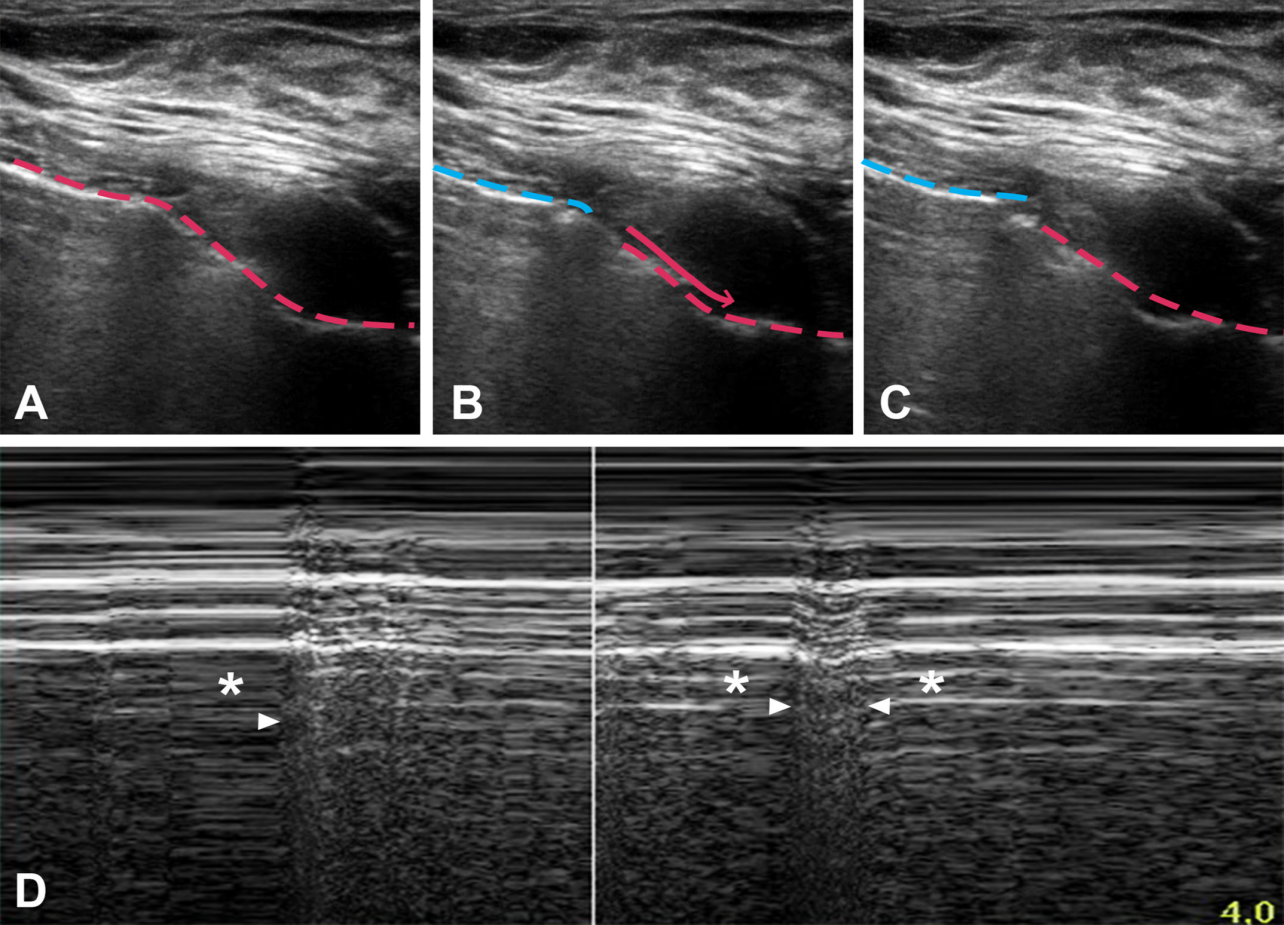
Typical lung ultrasound findings in patients with bullous emphysema: Bulla‐point and barcode sign. (A–C) Bulla‐point shown in B‐mode. Pictures were made at different points of the respiratory cycle: Rest (A), beginning of inspiration (B), end of inspiration (C). The pleuropulmonary interface is represented by a dotted line. During inspiration, a discontinuity occurs in the pleural line, as normal pleural sliding is observed in the caudal part of the image (red dotted line with arrow) while the cranial part shows comparatively much decreased pleural sliding (blue dotted line). A video version is presented in the Supporting Information (Video S1). (D) Barcode shown in TM mode (arrowheads). The asterisk indicates the beginning of a voluntary breath holding manoeuvre.

Compared to non‐bullous regions (430/504, 85.3% of all lung regions), bullous regions (74/504, 14.6%) demonstrated more frequent absence of lung sliding (33.7% vs. 11% in non‐bullous regions, *p* < 0.01), a barcode sign (14.9% vs. 2.6%, p < 0.01), increased A‐line visibility (16.2% vs. 8.4%, *p* = 0.048), and absence of Z lines (62.2% vs. 43.5%, *p* = 0.018). A bulla point was described in 3 different patients, in 3/74 (4.1%) bullous regions and 1/430 (0.2%) non‐bullous regions (*p* = 0.008) (Figure 2, Video S1).

**FIGURE 3:**
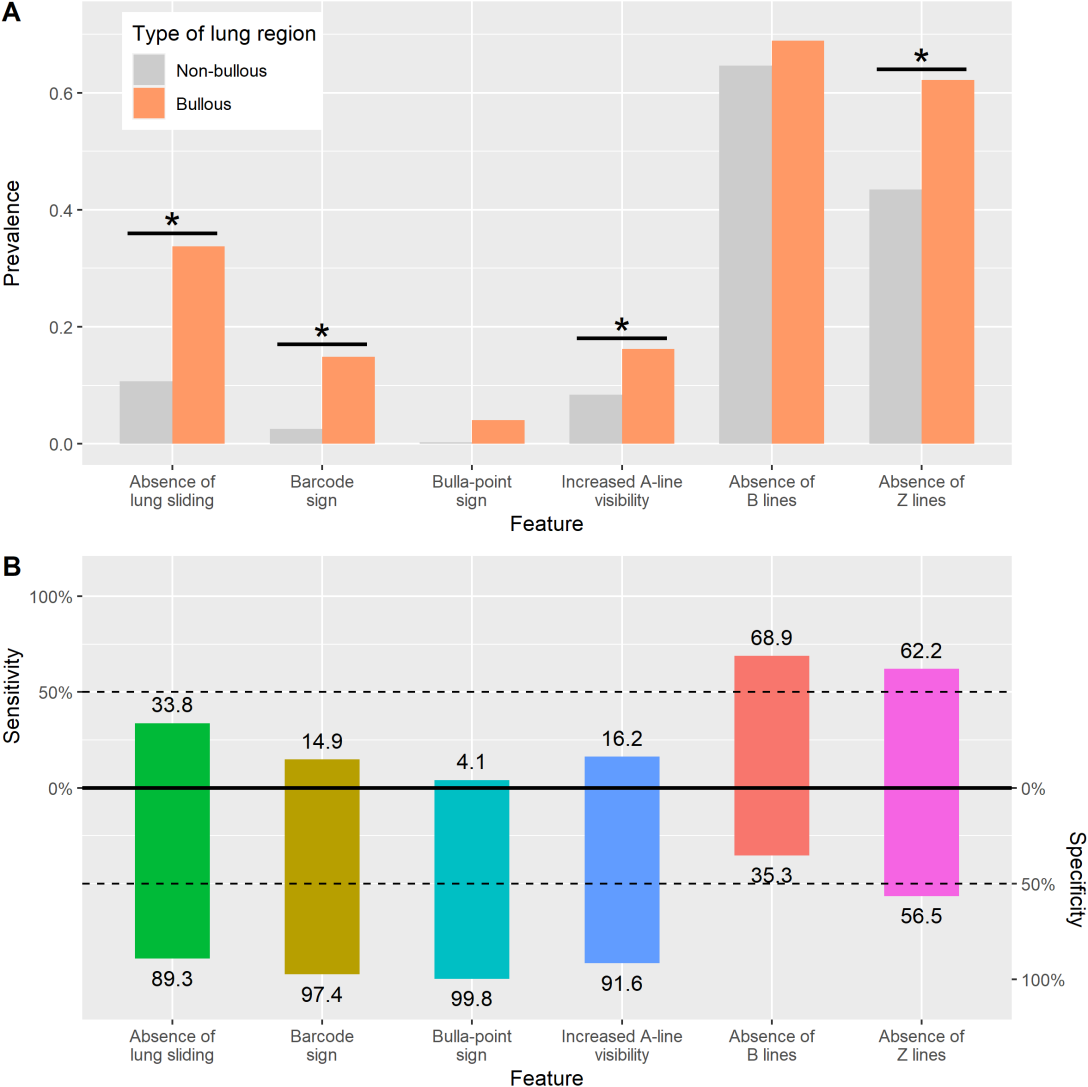
Comparison of LUS features between bullous and non‐bullous regions. (A) Bar plot of the prevalence of six sonographic features in regions with bullous emphysema with at least two intercostal spaces of pleural contact (bullous regions) and without (non‐bullous regions). Asterisks indicate statistical significance (*p* < 0.05). (B) Representation of the overall diagnostic performances of each sonographic feature for the positive diagnosis of the bullous region. Sensitivity is displayed on the upper part of the vertical axis, while specificity is shown on the lower part.

Features with the highest specificity for bullae were the absence of lung sliding (89.3%), the barcode sign (97.4%), the bulla‐point sign (99.8%) and the increase in A‐line visibility (91.6%). Prevalence and diagnostic performance (sensitivity and specificity) of LUS signs are reported in Figure 3.

### 3.3 Subgroup Analyses and Bulla Score

To account for the topographical heterogeneity in lung movement during respiration and the impact of pulmonary distension, subgroup analyses were conducted, separating apical regions (1 and 7) and non‐apical regions (2 to 6), as well as patients with pulmonary distension (TLC > 120%pred.). In these analyses, differences between bullous and non‐bullous regions remained statistically significant, and the loss of lung sliding was even more frequent in apical bullous regions (56.7% vs. 27.2% in apical non‐bullous regions) and in patients with pulmonary distension (52.6%). Barcode sign was also more frequently evidenced in apical regions compared to non‐apical regions (33.3% vs. 9.1% of bullous regions, respectively). Exhaustive LUS findings are reported in Table 3. Subgroup diagnostic performance analysis is reported in Table S1.

**TABLE 3:** Sonographic feature prevalence in bullous and non‐bullous regions.

Feature	Subgroup analysis	Bullous regions	Non‐bullous regions	Adjusted *p* value^a^
Absence of lung sliding	All lung regions	25/74 (33.8)	46/430 (10.7)	**< 0.001**
	Apical regions	17/30 (56.7)	31/114 (27.2)	**0.007**
	Non‐apical regions	8/44 (18.2)	15/316 (4.7)	**0.006**
	TLC > 120%	10/19 (52.6)	14/149 (9.4)	**< 0.001**
	TLC < 120%	11/40 (27.5)	23/212 (10.8)	**0.01**
Barcode sign	All lung regions	11/74 (14.9)	11/430 (2.6)	**< 0.001**
	Apical regions	7/30 (23.3)	5/114 (4.4)	**0.007**
	Non‐apical regions	4/44 (9.1)	6/316 (1.9)	**0.023**
	TLC > 120%	2/19 (10.5)	1/149 (0.7)	**0.024**
	TLC < 120%	6/40 (15)	7/212 (3.3)	**0.008**
Bulla‐point sign	All lung regions	3/74 (4.1)	1/430 (0.2)	**0.008**
	Apical regions	1/30 (3.3)	0/114 (0)	0.192
	Non‐apical regions	2/44 (4.5)	1/316 (0.3)	**0.031**
	TLC > 120%	2/19 (10.5)	1/149 (0.7)	**0.024**
	TLC < 120%	1/40 (2.5)	0/212 (0)	0.16
Increase in A line visibility	All lung regions	12/74 (16.2)	36/430 (8.4)	**0.034**
	Apical regions	5/30 (16.7)	11/114 (9.6)	0.189
	Non‐apical regions	7/44 (15.9)	25/316 (7.9)	0.075
	TLC > 120%	6/19 (31.6)	12/149 (8.1)	**0.008**
	TLC < 120%	3/40 (7.5)	5/212 (2.4)	0.113
Absence of B lines	All lung regions	51/74 (68.9)	278/430 (64.7)	0.213
	Apical regions	19/30 (63.3)	81/114 (71.1)	0.213
	Non‐apical regions	32/44 (72.7)	197/316 (62.3)	0.115
	TLC > 120%	13/19 (68.4)	95/149 (63.8)	0.324
	TLC < 120%	30/40 (75)	141/212 (66.5)	0.169
Absence of Z lines	All lung regions	46/74 (62.2)	187/430 (43.5)	**0.007**
	Apical regions	23/30 (76.7)	63/114 (55.3)	**0.034**
	Non‐apical regions	23/44 (52.3)	124/316 (39.2)	0.075
	TLC > 120%	12/19 (63.2)	51/149 (34.2)	**0.023**
	TLC < 120%	25/40 (62.5)	101/212 (47.6)	0.07

Abbreviation: TLC, total lung capacity (% of predicted value).

^a^ *p* values were adjusted for multiple comparison testing using the adaptive Benjamini‐Hochberg method with a false discovery rate set at 5%. Bolded values indicate statistical significance.

Signs included for constructing the bulla score were loss of lung sliding, barcode sign, bulla‐point, and increased A‐line visibility (+1 per sign per lung region). Total patient bulla score (3 [2–6]) was moderately correlated to bulla size (measured in intercostal spaces of pleural contact on chest CT): *r* = 0.53 [0.25;0.73], *p* < 0.001 (Figure S2). Interestingly, bulla score was also negatively correlated to FEV_1_% (*r* = −0.38 [−0.60;−0.03], *p* = 0.022) and FVC% (*r* = −0.38 [−0.64;−0.08], *p* = 0.021). There was a positive correlation to RV%pred. (*r* = 0.32 [−0.04;0.61] *p* = 0.081) and TLC%pred. (*r* = 0.17 [−0.30;0.43], *p* = 0.37), albeit not statistically significant (Figure S3).

## 4 Discussion

To the best of our knowledge, this is the first prospective multicentre study to focus on a precise and detailed description of the sonographic features of bullous emphysema. We demonstrated that commonly recognised signs of pneumothorax, such as absence of lung sliding, barcode sign, and increased A‐line visibility, are prevalent in bullous emphysema, especially in larger bullae, in patients with high pulmonary distension and in apical regions. We also confirmed the existence of the bulla‐point, a sonographic sign similar to the lung‐point of pneumothorax.

Chronic respiratory failure was frequent in our study population: one third of patients were on long‐term oxygen therapy and one sixth on home non‐invasive ventilation, slightly higher than previously reported figures in the COPD population at large [35]. The observed obstructive syndrome was often accompanied by distension and severe diffusion impairment, aligning with previous studies that found increased severity of ventilatory disorders in bullous emphysema [36], and showed that the size of bullae was inversely correlated to their participation in the ventilatory process [37]. Interestingly, 1 in 5 patients had a previous history of pneumothorax, underlining the risk of secondary pneumothorax in patients with bullous emphysema.

For a comprehensive description of bullous emphysema on LUS, we opted for a topographical segmentation of the thorax, coupled with a CT scan, the gold standard for evaluating emphysema [5]. Through this approach, we demonstrated that absent lung sliding and a barcode sign are frequently seen sonographic features of bullous emphysema, whereas lung sliding and the seashore sign are supposed to be near‐ubiquitous in healthy lung outside of acute exacerbations [38]. B lines were found in a third of bullous regions, similar to healthy lungs of patients of similar age [39]. On the other hand, increased A lines visibility was found in 16% of bullous regions, double the prevalence found in non‐bullous regions, and greater than expected in the lungs of elderly patients [39]. These horizontal lines are reverberation artefacts linked to the air‐tissue interface, representing the repetition of the pleuropulmonary line. In addition to being associated with pneumothorax, their visibility is known to be increased in exacerbating COPD patients, usually with conserved lung sliding [40]. The presence of this sign in our stable patients may point to the pathological nature of the pleuro‐pulmonary sonographic interface in bullous regions, as it comprises a layer of condensed, remodelled parenchyma (the bulla's wall) in addition to the two pleural sheets. In addition, subgroup analysis revealed an increase in the prevalence of these anomalies in the apical regions. This could be linked to the apical predominance of bullae and also to the regional ventilation specificities of the lung; indeed, lung expansion is greater in the lower parts of the lung compared to the apex, because of the influence of gravity and posture, potentially resulting in diminished lung sliding on LUS. Hyperinflation mechanisms associated with severe emphysema at large could also explain why we evidenced loss of lung sliding in a substantial proportion of non‐bullous regions, especially in the apex, which is in line with recently published research demonstrating frequent loss of lung sliding on POCUS of hyperinflated COPD patients [41].

The simple additive score we constructed using four criteria (absence of lung sliding, barcode sign, bulla‐point and increased visibility of A lines) was correlated to CT‐measured bulla size, which indicates that patients with the largest bullae had the most ultrasound abnormalities. Interestingly, these patients also had the most severe obstructive disorders and pulmonary distension, although these correlations were weaker. To improve on the evaluation of pulmonary distension using ultrasound and gain further insight into the respiratory mechanics in these patients, future studies would benefit from incorporating an evaluation of the diaphragmatic excursion, as patients with COPD have been shown to have increased diaphragm workload and impaired function, with reduced force reserve compared with healthy subjects [42].

Interestingly, we detected a bulla point sign in 3/36 (8.3%) patients with bullous emphysema, which confirms previous reports of signs mimicking the lung point in patients with bullae [24, 28]. Regions scored as positive for bulla point by investigators corresponded to an interruption in the pleuro‐pulmonary line, between an area of fully mobile lung and another area with decreased movement in B‐mode, resulting in a TM aspect of pseudo‐barcode. This sign should not be confused with the lung point, which is considered to be pathognomonic for pneumothorax and absent in healthy lung [33]. The complete immobility on one side of the “point” should be sought as it is the main difference between lung point and a bulla point, the latter of which exhibits some movement on both sides (albeit reduced on the bulla side) [33]. In addition, normal lung can also produce interruptions in the pleuro‐pulmonary line when observing lung fissures, with some differences: the two sides of the fissures move with respiration and the “interruption” is therefore mobile, with the addition of B lines in the discontinuous area (type 1 fissures) or an overlap between cranial and caudal regions (types 2 and 3) [43]. This observation highlights the need for clinicians confronted with such findings to attentively observe the transition area in B‐mode, and to seek small residual movement in regions appearing as immobile at first look, especially in emphysematous patients, so as to avoid confusing bulla and pneumothorax. This point is further underscored by a recent single‐centre study by the emergency room setting conducted by Karacabey et al. [44] The authors recruited patients presenting with dyspnoea, unilateral abolition of breathing sounds, and equivocal chest radiography for bullous emphysema or pneumothorax. In this CT‐controlled study, investigators recorded ultrasound findings patient‐to‐patient, with no segmentation of the lungs. Lung sliding appeared to have near‐perfect diagnostic performance for pneumothorax (Se 97.5%, Sp 100%), whereas, surprisingly, TM mode had abysmal performance as the authors described barcode signs in 100% of patients in both groups.

Overall, our results indicate that commonly recognised signs of pneumothorax, such as the absence of lung sliding, barcode sign, bulla‐point, and increased A‐line visibility, are also prevalent in bullous emphysema, especially in patients with high pulmonary distension or in apical regions. This overlap underscores the importance of careful interpretation of these signs in patients at risk of bullae or with severe chronic respiratory function impairment to prevent potential misdiagnosis. In the absence of prospective studies comparing LUS and CT in COPD patients with severe emphysema, we recommend that clinicians rely on CT imaging to confirm LUS signs of pneumothorax, as it remains the established gold standard for evaluating emphysema as well as pneumothorax.

Some limitations of our study should be noted; firstly, the relatively stringent inclusion criteria regarding bullae size and the exclusion of patients with acute organ failure may limit the generalisability of our findings. In addition, the size of our study population was relatively small; however, this could also be linked to the relative rarity of bullous emphysema and the heterogeneous recruitment of these patients, concentrating in expert centres with a specific focus on the management of chronic respiratory failure. Finally, CT scanners could be performed up to 2 years before inclusion and LUS. In practice, we observed a relatively short median interval of 14 days between CT and LUS, as the patients in the study population had often undergone recent imaging as part of their regular COPD follow‐up. This was still a potential source of bias, as patients at the time of LUS could in theory have slightly different CT presentations. We took this into account by requiring a chest radiography on the day of LUS to screen for confounding conditions such as pneumothorax and pleural effusions and excluding patients with acute respiratory failure. With regards to bullae, considering their irreversible nature, we do not believe such short time intervals significantly impacted their topographical descriptions.

In conclusion, through a rigorous, anatomically segmented approach, we provided a comprehensive description of the sonographic features of bullous emphysema. We found that some aspects of bullous emphysema, particularly in regions of extensive contact with the thoracic wall and poor ventilation, can mimic sonographic features traditionally associated with pneumothorax. These results suggest a lower specificity for these signs of pneumothorax in patients with COPD and bullous emphysema and emphasise the necessity for careful interpretation of ultrasound findings in clinical practice.

## Author Contributions

**Kinan El Husseini:** conceptualization (equal), data curation (equal), formal analysis (equal), investigation (equal), visualization (equal), writing – original draft (equal), writing – review and editing (equal). **Thomas Flament:** conceptualization (supporting), supervision (supporting), writing – review and editing (equal). **Sophie Laroumagne:** conceptualization (supporting), supervision (supporting), writing – review and editing (equal). **Damien Basille:** conceptualization (supporting), supervision (supporting), writing – review and editing (equal). **Mathilde Le Brun:** conceptualization (supporting), supervision (supporting), writing – review and editing (equal). **Elise Noël‐Savina:** conceptualization (supporting), supervision (supporting), writing – review and editing (equal). **Philippe Richard:** conceptualization (supporting), supervision (supporting), writing – review and editing (equal). **Gilles Mangiapan:** conceptualization (equal), methodology (equal), supervision (equal), writing – review and editing (equal). **Elise Artaud‐Macari:** conceptualization (equal), formal analysis (lead), methodology (lead), project administration (equal), supervision (lead), writing – review and editing (equal).

## Ethics Statement

The study design received approval from an ad‐hoc ethics committee (CPP, registry number 2019T3‐06 HPS [2018‐A00497‐48]) and was registered on the NIH ClinicalTrials.gov platform as an observational study (NCT04012359 registration date: 2019‐07‐09). Written informed consent to participate as well as consent for publication was obtained from all the participants of the study before enrolment. The research was conducted in accordance with the principles of the Declaration of Helsinki on clinical research involving human subjects.

## Conflicts of Interest

The authors declare no conflicts of interest.

## Supporting information


**Data S1.** Supporting Information.

**Video S1.** Images.

Video S1.

## Data Availability

Data used to perform this study are available upon request to the corresponding author.
