# Supplementary material for: Mapping Bullous Emphysema With Lung Ultrasound: A Prospective Multicentre Study
Source: Respirology. 2025 Mar 9;30(7):633–43. doi: 10.1111/resp.70021 (PMC12231765; doi:10.1111/resp.70021)
Supplement: Supplementary file 1 — Data S1. Supporting Information. [file RESP-30-633-s002.docx]

***Mapping Bullous Emphysema with Lung Ultrasound: A Prospective Multicentre Study***

**SUPPLEMENTARY MATERIAL**

**Supplementary Methods**

**Data collection in addition to lung ultrasound**

Institutional electronic health records at each centre were used to collect the following clinical data: month and year of birth, sex, tobacco exposure, past medical history (COPD, thoracic surgery, other respiratory diseases and any other notable history), date of COPD diagnosis, current medication, home oxygen and/or non-invasive ventilation prescription, pulmonary rehabilitation history, system by system clinical examination. The following paraclinical data were also collected: pulmonary function test results (weight, height, spirometry, plethysmography), chest computed tomodensitometry (CT) data : bullae topography (regions with pleural contact, number of intercostal spaces), emphysema characteristics (distribution, diffuse or localized), other parenchymal abnormalities (nodules, condensations, atelectasis, interstitial anomalies, cysts), pleural abnormalities (pneumothorax, pleural effusion, pleural thickening, pleural plaques), anomalies of the bone structure (scoliosis, vertebral fractures, costal fractures).

**CT image acquisition and analysis**

Chest CT examinations could have been performed in-centre or in another radiology department, and a standardized protocol was not required. Image quality was deemed sufficient if bullae walls could be seen and followed by the investigator, and intercostal spaces could be counted. Study investigators, who were senior pulmonologists, screened CT reports, reassessed all images, and measured the number of intercostal spaces in direct contact with bullous walls.

**Lung ultrasound machines**

Machines used to perform lung ultrasounds varied across centres. The following models were used (model [manufacturer]): Sonosite SII (FUJIFILM Europe), Logiq 9 (General Electric).

| **Supplementary Table S1.** Diagnostic performances of sonographic features for identifying bullae. | | | | | |
| --- | --- | --- | --- | --- | --- |
| **Variables** | **All lung regions** | **Apical**  **regions** | **Non-apical**  **regions** | **TLC**  **> 120%** | **TLC**  **< 120%** |
| **Absence of lung sliding** | | | | | |
| Bullous regions, n (%) | 25/74 (33.8) | 17/30 (56.7) | 8/44 (18.2) | 10/19 (52.6) | 11/40 (27.5) |
| Non-bullous regions, n (%) | 46/430 (11) | 31/114 (27) | 15/316 (4.7) | 14/149 (9.4) | 23/212 (10.8) |
| Se (%) | 33.8 | 56.7 | 18.2 | 52.6 | 27.5 |
| Sp (%) | 89.3 | 72.8 | 95.3 | 90.6 | 89.2 |
| PPV (%) | 35.2 | 35.4 | 34.8 | 41.7 | 32.4 |
| NPV (%) | 88.7 | 86.5 | 89.3 | 93.8 | 86.7 |
| **Barcode sign** | | | | | |
| Bullous regions, n (%) | 11/74 (14.9) | 7/30 (23.3) | 4/44 (9.1) | 2/19 (10.5) | 6/40 (15) |
| Non-bullous regions, n (%) | 11/430 (2.6) | 5/114 (4.4) | 6/316 (1.9) | 1/149 (0.7) | 7/212 (3.3) |
| Se (%) | 14.9 | 23.3 | 9.1 | 10.5 | 15.0 |
| Sp (%) | 97.4 | 95.6 | 98.1 | 99.3 | 96.7 |
| PPV (%) | 50.0 | 58.3 | 40.0 | 66.7 | 46.2 |
| NPV (%) | 86.9 | 82.6 | 88.6 | 89.7 | 85.8 |
| **Bulla-point sign** | | | | | |
| Bullous regions, n (%) | 3/74 (4.1) | 1/30 (3.3) | 2/44 (4.5) | 2/19 (10.5) | 1/40 (2.5) |
| Non-bullous regions, n (%) | 1/430 (0.2) | 0/114 (0) | 1/316 (0.3) | 1/149 (0.7) | 0/212 (0) |
| Se (%) | 4.1 | 3.3 | 4.5 | 10.5 | 2.5 |
| Sp (%) | 99.8 | 100.0 | 99.7 | 99.3 | 100.0 |
| PPV (%) | 75.0 | 100.0 | 66.7 | 66.7 | 100.0 |
| NPV (%) | 85.8 | 79.7 | 88.2 | 89.7 | 84.5 |
| **Increase in A line visibility** | | | | | |
| Bullous regions, n (%) | 12/74 (16.2) | 5/30 (16.7) | 7/44 (15.9) | 6/19 (31.6) | 3/40 (7.5) |
| Non-bullous regions, n (%) | 36/430 (8.4) | 11/114 (9.6) | 25/316 (7.9) | 12/149 (8.1) | 5/212 (2.4) |
| Se (%) | 16.2 | 16.7 | 15.9 | 31.6 | 7.5 |
| Sp (%) | 91.6 | 90.4 | 92.1 | 91.9 | 97.6 |
| PPV (%) | 25.0 | 31.2 | 21.9 | 33.3 | 37.5 |
| NPV (%) | 86.4 | 80.5 | 88.7 | 91.3 | 84.8 |
| **Absence of B lines** | | | | | |
| Bullous regions, n (%) | 51/74 (68.9) | 19/30 (63.3) | 32/44 (72.7) | 13/19 (68.4) | 30/40 (75) |
| Non-bullous regions, n (%) | 278/430 (65) | 81/114 (71) | 197/316 (62) | 95/149 (64) | 141/212 (67) |
| Se (%) | 68.9 | 63.3 | 72.7 | 68.4 | 75.0 |
| Sp (%) | 35.3 | 28.9 | 37.7 | 36.2 | 33.5 |
| PPV (%) | 15.5 | 19.0 | 14.0 | 12.0 | 17.5 |
| NPV (%) | 86.9 | 75.0 | 90.8 | 90.0 | 87.7 |
| **Absence of Z lines** | | | | | |
| Bullous regions, n (%) | 46/74 (62.2) | 23/30 (76.7) | 23/44 (52.3) | 12/19 (63.2) | 25/40 (62.5) |
| Non-bullous regions, n (%) | 187/430 (43) | 63/114 (55) | 124/316 (39) | 51/149 (34) | 101/212 (48) |
| Se (%) | 62.2 | 76.7 | 52.3 | 63.2 | 62.5 |
| Sp (%) | 56.5 | 44.7 | 60.8 | 65.8 | 52.4 |
| PPV (%) | 19.7 | 26.7 | 15.6 | 19.0 | 19.8 |
| NPV (%) | 89.7 | 87.9 | 90.1 | 93.3 | 88.1 |
| Abbreviations: TLC: total lung capacity (% of predicted value); Se: sensitivity; Sp: specificity; PPV: positive predictive value; NPV: negative predictive value. | | | | | |

**
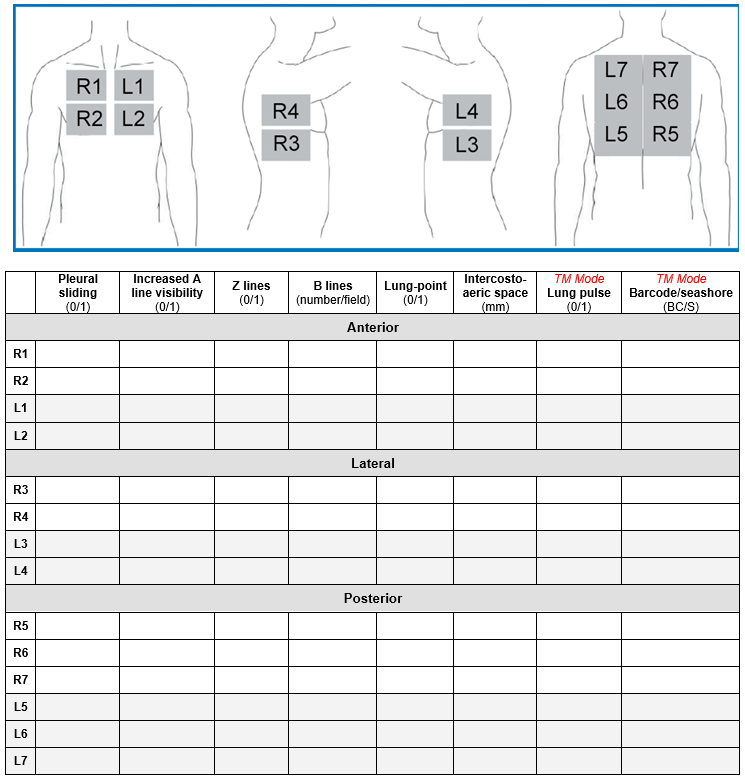
**

**Supplementary Figure S1.** Translated version of the standardized form used to record ultrasound findings.


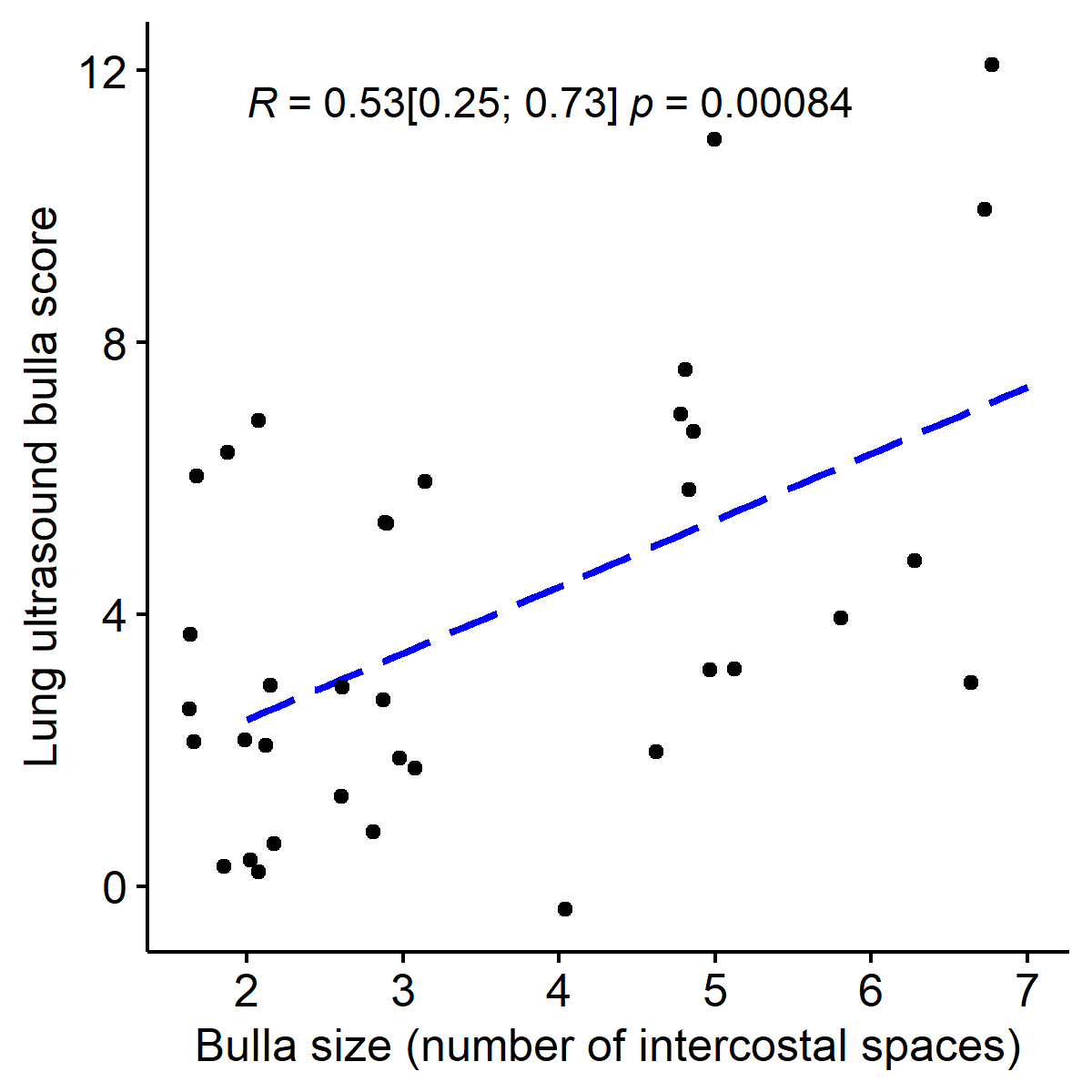


**Supplementary Figure S2. Scatter-plots of patient ultrasound bulla score against bulla size**. Bulla size was measured as number of intercostal spaces of pleural contact on chest CT. Bulla score: every lung region was scored according to 4 criteria: absence of lung sliding, barcode sign, bulla-point sign, A-line visibility (score of 0 to +4 for each region). The sum total of scores in every lung region constituted the bulla score for each patient. Abbreviations: R: Pearson r statistic (with confidence interval); p: p-value.


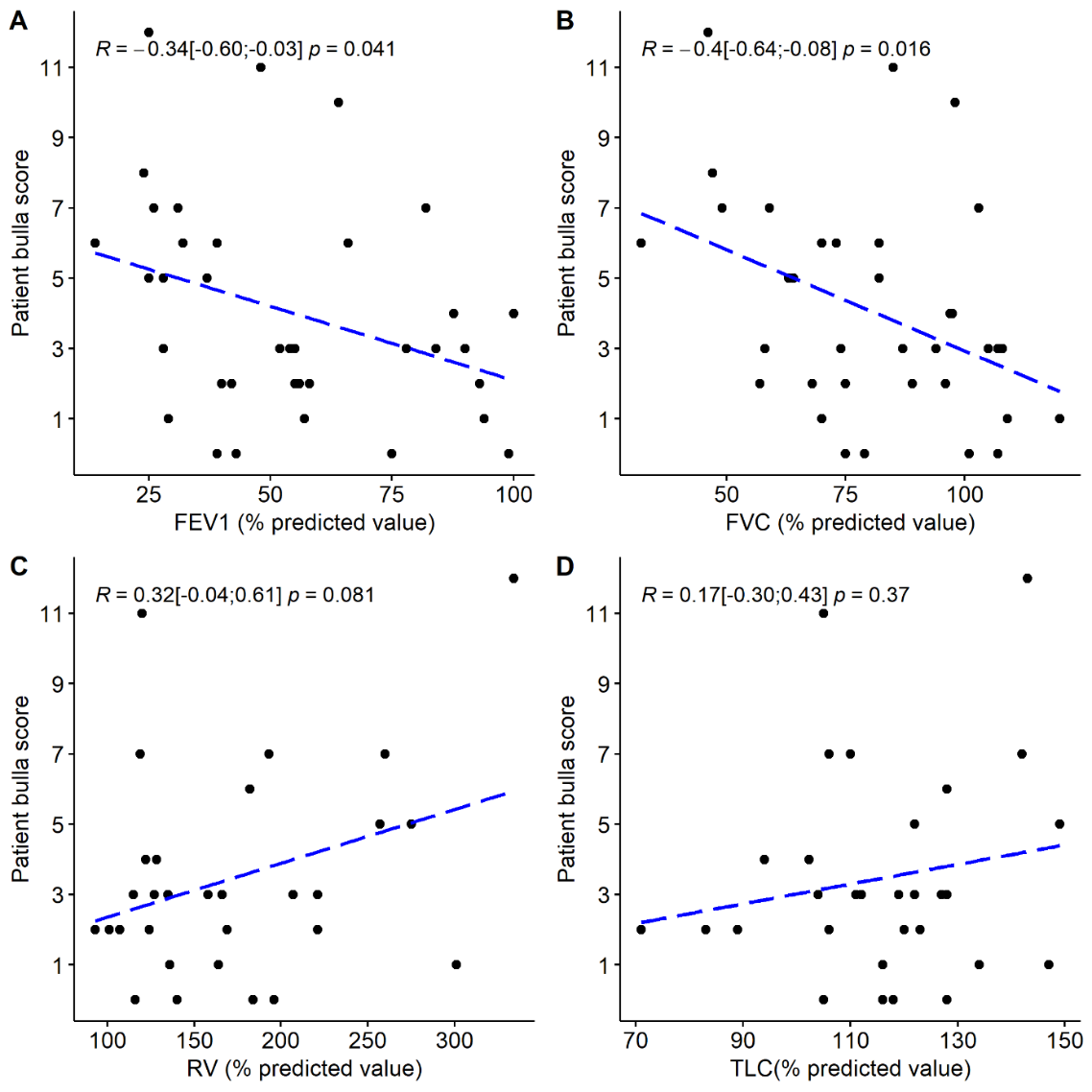


**Supplementary Figure S3.** Scatter-plots of patient bulla score* against respiratory variables, with linear regression line. **(A)** Bulla score against forced maximum expiratory volume in 1 second (FEV1) (% of predicted value). **(B)** Bulla score against forced viral capacity (FVC) (% of predicted value). **(C)** Bulla score against residual volume (% of predicted value). *Bulla score: every lung region was scored according to the presence of sonographic features found to be specific for bullous emphysema with pleural contact (specificity > 85%) (score of 0 to +4 for each region). The sum total of scores in every lung region constituted the bulla score for each patient. Abbreviations: R: Pearson r statistic (with confidence interval); p: p-value.

**Supplementary Video S1.** Bulla point observed on lung ultrasound (B mode) in a patient with bullous emphysema. During inspiration, a discontinuity is visible in the pleural line. Pleural sliding with a normal amplitude is observed in the caudal region (right side) while the cranial part shows comparatively much decreased pleural sliding. This video was captured in the antero-inferior region (R2), which corresponded to a large bullous formation on CT.
