# Supplementary figures and images for: Mapping Bullous Emphysema With Lung Ultrasound: A Prospective Multicentre Study
Source: Respirology. 2025 Mar 9;30(7):633–43. doi: 10.1111/resp.70021 (PMC12231765; doi:10.1111/resp.70021)

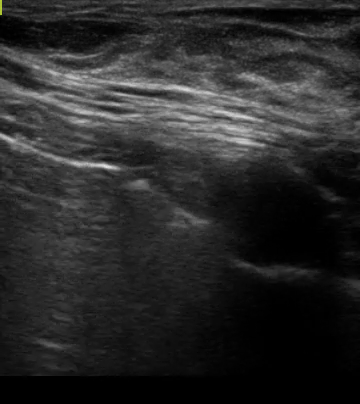

Supplement: Supplementary file 2 — Video S1. Images. [file RESP-30-633-s003.png]
